# ADMINISTRATIVE INDICATORS OF FAMILY SUPPORT AMONG COMPLEX PATIENTS

**DOI:** 10.1093/geroni/igz038.800

**Published:** 2019-11-08

**Authors:** Elvira E Jimenez, Ranak Trivedi, Alexis Huynh, Taona Haderlein, Marian Katz, Evelyn Chang

**Affiliations:** 1 Center for the Study of Healthcare Innovation, Implementation & Policy (CSHIIP), V.A. Greater Los Angeles Healthcare System, Los Angeles, California, United States; 2 Center for Innovation to Implementation, VA Palo Alto Health Care System, Menlo Park, California, United States; 3 Center for the Study of Healthcare Innovation, Implementation & Policy (CSHIIP), V.A. Greater Los Angeles Healthcare System, Los Angeles, CA, Los Angeles, California, United States

## Abstract

Greater access to family support has been shown to positively affect the management of complex patients (i.e., multiple chronic conditions and psychosocial needs). However, patients’ availability of family support is not easily obtainable from medical records. We aim to identify administrative variables that can be used as indicators of family support. We investigated secondary next-of-kin (i.e., patient identified two next-of-kin) and marital status as administratively defined family support availability in a Veteran sample (n=2210). A subsample (n=329) was further evaluated with documented responses to questions “Are there any friends/family members you would like to involve in any aspect of your health care?” (i.e., “actual availability”) and “Does anyone help you with your daily activities?” (i.e., “obtained” availability). We performed a logistic regression analysis to evaluate the association between administratively defined and “actual” or “obtained” availability of support controlling for age, race/ethnicity, and gender. The sample was 90% male, mean age 63 years, 50%White and 44% African American. We found that 32.9% had administratively defined availability by being married, and 32.5% by listing secondary next-of kin. Married Veterans were significantly more likely to report greater actual availability (p=0.01) and obtained (p=0.04) support. Veterans listing a secondary next-of-kin were significantly more likely to report “actual” availability (p= 0.04) but not on “obtained” (p=0.08) support. Marital status may be a useful proxy of actual family support and listing a secondary next-of kin may be an alternate indicator for complex patients. Our study provides guidance on the use of administrative data in understanding caregivers.

